# Hippo signaling pathway and respiratory diseases

**DOI:** 10.1038/s41420-022-01020-6

**Published:** 2022-04-20

**Authors:** Weifeng Tang, Min Li, Xiaoting Yangzhong, Xifeng Zhang, Anju Zu, Yunjiao Hou, Lin Li, Shibo Sun

**Affiliations:** 1 Department of Pulmonary and Critical Care Medicine, First Affiliated Hospital, Kunming Medical University, Kunming, China; 2 Clinical Medicine, Seven class, 2019 Grade, Kunming Medical University, Kunming, China; 3 Pediatrics, First class, 2020 Grade, Kunming Medical University, Kunming, China

**Keywords:** Molecular biology, Cell biology

## Abstract

The hippo signaling pathway is a highly conserved evolutionary signaling pathway that plays an important role in regulating cell proliferation, organ size, tissue development, and regeneration. Increasing evidences consider that the hippo signaling pathway is involved in the process of respiratory diseases. Hippo signaling pathway is mainly composed of mammalian STE20-like kinase 1/2 (MST1/2), large tumor suppressor 1/2 (LATS1/2), WW domain of the Sav family containing protein 1 (SAV1), MOB kinase activator 1 (MOB1), Yes-associated protein (YAP) or transcriptional coactivator with PDZ-binding motif (TAZ), and members of the TEA domain (TEAD) family. YAP is the cascade effector of the hippo signaling pathway. The activation of YAP promotes pulmonary arterial vascular smooth muscle cells (PAVSMCs) proliferation, which leads to pulmonary vascular remodeling; thereby the pulmonary arterial hypertension (PAH) is aggravated. While the loss of YAP leads to high expression of inflammatory genes and the accumulation of inflammatory cells, the pneumonia is consequently exacerbated. In addition, overexpressed YAP promotes the proliferation of lung fibroblasts and collagen deposition; thereby the idiopathic pulmonary fibrosis (IPF) is promoted. Moreover, YAP knockout reduces collagen deposition and the senescence of adult alveolar epithelial cells (AECs); hence the IPF is slowed. In addition, hippo signaling pathway may be involved in the repair of acute lung injury (ALI) by promoting the proliferation and differentiation of lung epithelial progenitor cells and intervening in the repair of pulmonary capillary endothelium. Moreover, the hippo signaling pathway is involved in asthma. In conclusion, the hippo signaling pathway is involved in respiratory diseases. More researches are needed to focus on the molecular mechanisms by which the hippo signaling pathway participates in respiratory diseases.

## Facts

- Hippo signaling pathway, as a highly conserved evolutionary pathway, plays an important role in regulating cell proliferation, organ size, tissue development, and regeneration.
- Accumulative evidences suggest that the hippo signaling pathway is involved in respiratory diseases.
- YAP promotes PAVSMCs proliferation and pulmonary vascular remodeling via PI3K/AKT pathway; thereby PAH is aggravated.
- When the stiffness of the extracellular matrix increases, YAP enters the nucleus and binds to TEAD to promote Twist1 gene transcription, cell proliferation, and collagen deposition, which cause the pulmonary fibrosis.

## Open questions

- What are the core components of the hippo signaling pathway in cells?
- How the hippo signaling pathway plays the functions in cells?
- What are the mechanisms that the hippo signaling pathway regulates respiratory diseases?

## Introduction

The hippo signaling pathway is first identified in Drosophila melanogaster during screening for genes that negatively regulate tissue growth and is a highly conserved evolutionary signaling pathway that plays a central role in controlling tissue homeostasis, development, regeneration, and organ size through the regulation of cell proliferation and apoptosis [1–3]. Increasing studies confirm that the hippo signaling pathway is involved in regulating a variety of physiological processes in the human body, and its dysfunction leads to uncontrolled cell growth and even malignant transformation [4–8]. In particular, the hippo signaling pathway plays an irreplaceable role in regulating tumor initiation, tumor propagation, tumor resistance to therapy, innate immunity, and adaptive immunity [4–8]. Nevertheless, accumulative evidences suggest that the hippo signaling pathway is closely related to respiratory diseases such as acute lung injury (ALI), pneumonia, idiopathic pulmonary fibrosis (IPF), pulmonary arterial hypertension (PAH), asthma, etc. [9–11]. This article aimed to review the mechanisms of the hippo signaling pathway involvement in respiratory diseases.

## Hippo signaling pathway

### Core components of the hippo signaling pathway

The core components of the hippo signaling pathway consist of a kinase cascade transcription of the upstream and effector factors of the downstream [12, 13]. The kinase cascades of hippo signaling pathway in mammalian cells mainly include mammalian STE20-like kinase 1/2 (MST1/2), large tumor suppressor 1/2 (LATS1/2), WW domain of Sav family containing protein 1 (SAV1), and MOB kinase activator 1 (MOB1) [3, 12–14]. Hippo kinase cascades initiated by thousand-and-one amino acid kinase (TAOK), which phosphorylates the activation loop of MST1/2, thereby activating MST1/2 [15]. Also, MST1/2 is activated by autophosphorylation of the MST dimer activation loop [15, 16]. The carboxyl terminal of MST1/2 has a distinctive coiled-coil structure which is called the Sav/RassF/Hpo (SARAH) domain [16]. The activated MST1/2 is heterodimerized with SAV1 through c-terminal SARAH domain to form the MST1/2-SAV1 complex [17, 18]. The MST1/2-SAV1 complex recruits LATS1/2 and binds MST1/2 to LATS1/2 [12, 15, 19]. Subsequently, MST1/2 phosphorylates the hydrophobic motif (HM) of LATS1/2 [20, 21]. With the assistance of MOB1, phosphorylated HM triggers autophosphorylation of LATS1/2 in the activation loop; thus the kinases fully are activated [20, 21]. In addition, other kinases parallel to MST1/2 including mitogen-activated protein kinase kinase kinase kinases 1–7 (MAP4K1-7) and TAOK1-3, can also directly phosphorylate the HM of LATS1/2, leading to activation of LATS1/2 [15, 18, 19, 22].

**Fig. 1:**
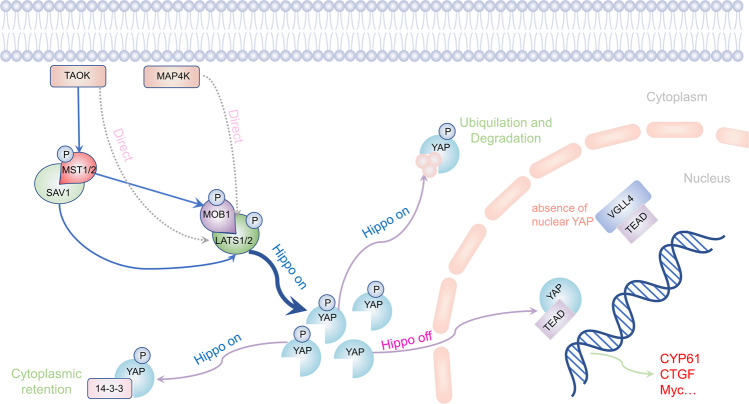
Hippo signaling pathway. The hippo pathway is mainly composed of MST1/2, LATS1/2, SAV1, MOB1, YAP or TAZ, and TEAD. When the hippo pathway is unactivated, unphosphorylated YAP enters the nucleus and binds to TEAD, thus inducing transcription of target genes. When the hippo pathway is activated, TAOK phosphorylates MST1/2. Phosphorylated MST1/2 binds to SAV1 to form the MST1/2-SAV1 complex. With the assistance of activated MOB1, the MST1/2-SAV1 complex induces phosphorylation of the LATS1/2. Phosphorylated LATS1/2 activates YAP, resulting in YAP being captured by 14-3-3 proteins in the cytoplasm or being degraded by SCFβ-TRCP E3 ubiquitin ligase mediated ubiquitin-proteasome pathway. Note: TAOK, thousand-and-one amino-acid kinase; MAP4K1-7 mitogen-activated protein kinase kinase kinase kinases 1–7, MST1/2 mammalian STE20-like kinase 1/2, SAV1 sav family containing protein 1, LATS1/2 large tumor suppressor 1/2, MOB1 MOB kinase Activator 1, TEA TEA domain family, VGLL4 vestigial-like family member 4.

Yes-associated protein (YAP)/transcriptional coactivator with PDZ-binding motif (TAZ) is the downstream effector of hippo signaling pathway and regulates the expression of target genes [3, 12]. When hippo signaling pathway is activated, activated LATS1/2 phosphorylates YAP at five sites (Ser61, Ser109, Ser127, Ser164, and Ser381) and TAZ at 4 four (Ser66, Ser89, Ser117, and Ser311) with a consensus phosphorylation motif of HxRxxS [16, 17, 19, 23]. The two amino acid residues most associated with YAP and TAZ degradation are Ser127 and Ser381 in YAP and Ser89, and Ser311 in TAZ [23]. Phosphorylation of Ser127 in YAP or Ser89 in TAZ creates a binding consensus for 14-3-3 proteins that sequesters YAP/TAZ in the cytoplasm [19, 23]. Both phosphorylation of Ser381 in YAP and Ser311 in TAZ trigger a sequential phosphorylation of casein kinase 1 (CK1), leading to recruitment of SCFβ-TRCP E3 ligase, ubiquitination, and proteasome degradation of YAP or TZA [19, 23]. When the hippo signaling pathway is unactivated, unphosphorylated YAP/TAZ transfers from the cytoplasm into the nucleus and binds to TEAD1-4, which has a DNA binding domain and a YAP/ TAZ binding domain [12, 15, 24]. The YAP/TAZ-TEAD protein complex regulates the expression of target genes such as connective tissue growth factor (CTGF), cysteine-rich angiogenic inducer 61 (CYR61), fibroblast growth factor (FGF1), and neuropeptide-1 (NRP1), which promote the cell growth and proliferation [19, 25–27]. In the absence of nuclear YAP/TAZ, TEAD combines with vestigial-like family member 4 (VGLL4) to form a default repression complex which acts as a transcription repressors [12, 15, 18] (Fig. 1).

### Upstream signals of the hippo signaling pathway

The hippo signaling pathway is regulated by G-protein-coupled receptors, mechanical cues, cell adhesion, cell polarity link-related proteins, and cell energy state [4, 28, 29].

G-protein-coupled receptor (GPCR) and its related ligands regulate YAP activity and the expression of YAP by regulating LATS1/2 kinase [30, 31]. Studies confirm that different GPCRs have different biological effects on YAP [25, 32]. GPCRs coupled with Gα12/13, Gα q /11, or Gα i/o promote the activation of YAP. On the contrary, GPCRs binding to Gαs inhibits YAP activation [25, 32].

In addition, the hippo signaling pathway is regulated by mechanical cues such as cell density, mechanical tension, and extracellular matrix stiffness [20]. In different cell density, the hippo signaling pathway shows different effects. In high-density cells, the activated hippo signaling pathway induces phosphorylation of YAP and the phosphorylation of YAP is located in the cytoplasm; thus the transcription of target genes and cell proliferation are inhibited [33, 34]. On the contrary, the hippo signaling pathway is not activated and YAP is not phosphorylated in low cell density [33, 34]. In addition, YAP bound to TEAD is located in the nucleus to induce the expression of target genes and promote cell proliferation [33]. Meanwhile, filamentous actin (F-actin) which response dynamically to mechanical changes, is one of the important regulatory factors of hippo signaling pathway [20]. When F-actin is broken down or lost, STK25 in the GCKIII kinase family is involved in various cell proliferation, transformation, migration, polarity, and apoptosis. Activated STK25 promotes the hippo signaling pathway activation by directly activating LATS1/2 [35–37]. On the contrary, the accumulation of F-actin inhibits the hippo signaling pathway by inhibiting the phosphorylation of LATS1/2 [35–37]. Moreover, when cells grow on the extracellular matrix with low stiffness, GTPase RAP2, MAP4K4, MAP4K6, and MAP4K7 activate the LATS1/2 and promote the degradation of YAP in the cytoplasm [38, 39]. While, when cells grow on the extracellular matrix with high stiffness, YAP locates in the nucleus and binds to TEAD transcription factors to promote expression of fibrosis genes and proliferation of cells [38, 39]. Consequently, the hippo signaling pathway plays an important role in sensing the mechanical microenvironment.

Additionally, it is reported that cell adhesion inhibits LATS1/2 by stimulating the FAK-SRC-PI3K-PDK1 pathway and then induces YAP transferring into the nucleus [40]. Accordingly, cellular adhesion may be a negative upstream regulator of the hippo signaling pathway.

Moreover, proteins involved in cell polarity and cell connection may play an important role in regulating the hippo signaling pathway [41–45]. By their WW domains, kidney and brain protein (KIBRA) is bound to Merlin, which is also known as neurofibromin 2 (NF2). Subsequently, the combination of KIBRA and Merlin recruits LATS1/2 to the cell membrane [41–43]. Meanwhile, the WW domain of the KIBRA binding to SAV results in the form of KIBRA/SAV heterodimer, which recruits MST1/2 to the cell membrane; thus the LATS1/2-HM is phosphorylated. Subsequently, phosphorylation of Ser127 in YAP interacts with 14-3-3 proteins to form a complex that remains in the cytoplasm, and the expression of target gene is also decreased [41–43]. It is reported angiomotin (AMOT) is associated with cell polarity, regulation of angiogenesis, cell migration, actin dynamics, and interaction with YAP [44, 45]. The AMOT interacts with F-actin through N-terminal of AMOT and promotes the stability of AMOT [20, 46–48]. However, when F-actin is destroyed, AMOT is phosphorylated by LATS1/2 and then binds to MST1/2, LATS1/2, SAV1, and YAP [20, 46–48]. In addition, AMOT can act as a Merlin binding scaffold protein, and the phosphorylation of Ser176 induces AMOT-YAP-Merlin complex to translocation from cytoplasm and nucleus to the plasma membrane, thereby YAP activity is affected [49]. Meanwhile, the interaction between AMOT and MOB1 promotes the autophosphorylation of LATS1/2 on the activated loop independent of HM phosphorylation [20].

Additionally, when cells suffer from energy starvation, AMPK activates and directly phosphorylates the amino acid residues of Ser94 in YAP, which inhibits YAP binding to TEAD, and then the YAP is inhibited [50]. Moreover, AMPK can directly inhibit the activation of YAP and promote YAP phosphorylating LATS1/2 through the phosphorylation of AMOTL1 [51].

## The hippo signaling pathway and respiratory diseases

### Hippo signaling pathway and ALI

ALI is characterized by the damage of alveolar epithelial cells and pulmonary capillary endothelial caused by noncardiogenic factor and results in acute hypoxic respiratory insufficiency [52, 53]. Severe ALI leads to life-threatening respiratory failure with high morbidity and mortality [54, 55]. Infiltration of inflammatory cells, increased blood-air barrier permeability, pulmonary edema, and diffuse alveolar damage are often found in ALI [54, 56, 57]. Meanwhile, excessive lung inflammation and apoptosis of alveolar epithelial cells (AECs) are key factors in the pathogenesis of ALI [52, 56].

The respiratory epithelial cells of mature lung are stationary under normal physiological conditions, but a variety of epithelial cells, such as type II alveolar epithelial cells (AECIIs) and basal cells, significantly regenerate when lung is injured [58–60]. It is reported that the YAP improves the self-renewal of AECIIs and the differentiation of AECIIs into type I alveolar epithelial cells (AECIs) with lung injury [59, 61, 62]. In addition, YAP is essential for AECIIs proliferating or differentiating into AECIs in response to mechanical tension [59, 61, 63, 64]. In the process of alveolar regeneration, AECIIs responds to the increase of mechanical forces outside the environment, which lead to the aggregation and the activation of YAP in nucleus and promote the proliferation of AECIIs as well as the differentiation of the AECIIs into AECIs [59, 61, 63, 64]. In addition, lung microvascular endothelial cells (LMVECs) release sphingosine-1-phosphate (S1P), which plays an important role in regulating the progenitor function of AECIIs during the repair of alveolar epithelial [65]. As a family of GPCRs, S1P receptor 2 (S1PR2) is affected by S1P and inhibits LATS1/2 through G12/13, which induce unphosphorylated YAP to enter the nucleus and subsequently mediate transcriptional expression of target genes, promote proliferation and differentiation of AECIIs [65]. Meanwhile, the loss of MST1/2 and the expression of YAP target Ajuba LIM protein which controls proliferation and differentiation of lung epithelial progenitor cells during lung repair [60, 61, 63]. Accordingly, the hippo signaling pathway may be involved in the repair process of ALI by improving proliferation and differentiation of lung epithelial progenitor cells.

The basal stem/progenitor cells (BSCs) are activated and recruited to the site of injury in lung, where they help to regenerate the lung epithelium [66]. Differentiated epithelial cells recruit integrin-linked kinases to adhesion sites with the injury of epithelial cells, which result in degradation of Merlin, downregulation of the hippo pathway, and induction of Wnt7b secretion [66, 67]. The Wnt7b induces airway smooth muscle cells (ASMCs) to release fibroblast growth factor 10 (Fgf10), which binds to fibroblast growth factor receptor 2 (Fgfr2b) on BSCs to mobilize and amplify stem/progenitor cell populations by inhibiting premature differentiation of BSCs, thereby promoting efficient lung regeneration [66, 67].

Moreover, alveolar epithelial cells are repaired by exogenous bone marrow-derived mesenchymal stem cells (BMSCs) and down-expression of LATS2 improves BMSCs repairing the tissue in ALI, thereby alleviates the pathological damage of lung tissue [57]. In addition, inhibition of hippo signaling pathway increases BMSCs retention and migration to the site of injured lung tissue and promoted the differentiation of BMSCs into AECIIs [57].

**Fig. 2:**
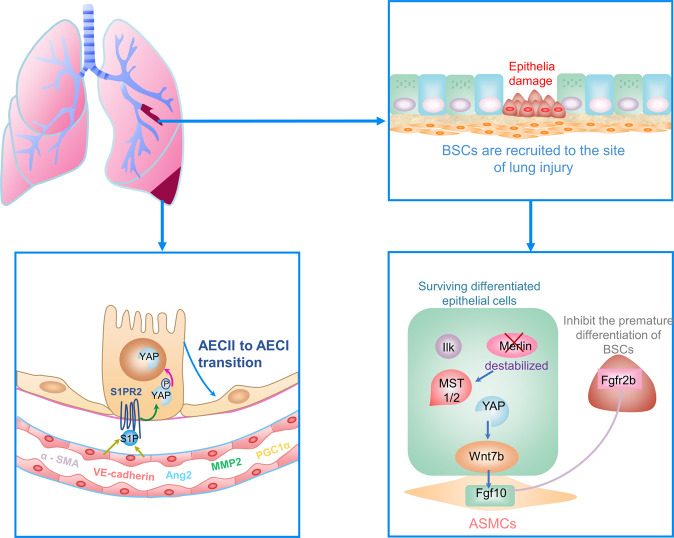
The role of hippo signaling pathway in ALI. During epithelial damage, BSCs are activated and recruited to the site of lung injury. Surviving differentiated epithelial cells recruit integrin-linked kinases to adhesion sites with the injury, which result in degradation of Merlin, downregulation of the hippo pathway, and the secretion of Wnt7b. The Wnt7b induces ASMCs to release Fgf10, which binds to Fgfr2b on BSCs to mobilize and amplify stem/progenitor cell populations by inhibiting premature differentiation of BSCs, thereby promoting lung regeneration. In addition, S1P released by LMVECs promotes the proliferation and differentiation of AECIIs through the S1P-S1PR2-YAP signaling axis, thus regulating alveolar epithelial repair. Meanwhile, YAP promotes the germination and remodeling of neovascules through various angiogenesis factors such as Ang2, MMP2, VE-cadherin, α‐SMA, and PGC1α. Note: ALI acute lung injury; BSCs basal stem/progenitor cells, ASMCs airway smooth muscle cells, Fgf10 fibroblast growth factor 10, Fgfr2b fibroblast growth factor receptor 2, S1P sphingosine-1-phosphate, LMVECs lung microvascular endothelial cells, AECIIs type II alveolar epithelial cells, AECI type I alveolar epithelial cells, YAP Yes-associated protein, Ang2 Angiopoietin-2, MMP2 matrix metallopeptidase 2, α‐SMA α-smooth muscle actin, PGC1α peroxisome proliferator-activated receptor γ coactivator 1α.

In addition, the repair of pulmonary capillary endothelial is important for ALI. YAP promotes the germination and remodeling of neovascules through various angiogenesis factors such as Ang2, MMP2, VE-cadherin, α‐SMA, and PGC1α [68, 69]. Knockout of the YAP reduces vascular density, budding, and branching [70]. In addition, it is reported that mutation of YAP significantly promoted alveolar budding [69]. Meanwhile, mechanical stimulation to the tissue microenvironment controls vascular morphogenesis and barrier function [71]. Mechanical tension, cell density, and angiogenic factor expression are different after pneumonectomy treatment [61, 63, 64, 69]. YAP activity is controlled by a variety of mechanical stimuli such as cell density, extracellular matrix stiffness, mechanical tension, etc. [72–74]. Inhibition of YAP activity can eliminate the potential of angiogenesis [75]. Accordingly, the hippo signaling pathway may be involved in the repair process of ALI by intervening in the repair of pulmonary capillary endothelial (Fig. 2).

### Hippo signaling pathway and PAH

PAH is a chronic, progressive pulmonary vascular disease with abnormally elevated pulmonary arterial pressure [76, 77].

Pathogenesis of PAH is associated with pulmonary artery cell proliferation, vascular remodeling, increased anti-apoptosis, and orthotopic thrombosis [78, 79]. Vascular remodeling caused by abnormal proliferation and impaired apoptosis of pulmonary arterial vascular smooth muscle cells (PAVSMCs) plays a key role in PAH [78, 80]. Increasing studies suggest that YAP of the hippo signaling pathway is involved in pulmonary vascular remodeling [78, 80]. The inactivation of LATS1 enhances the activity of YAP, which results in the proliferation of PAVSMCs and pulmonary vascular remodeling [78, 80]. In addition, YAP directly promotes the transcription of Pik3cb which encodes the catalytic subunit P110β of PI3K and enhances TEAD, thereby activating the PI3K/AKT pathway, and then the activation of PI3K/AKT pathway inhibits AKT phosphorylation to improve PAVSMCs proliferation and weaken pulmonary vascular remodeling [78]. Moreover, inactivation of LATS1 is caused by negative bidirectional cross-linking between YAP-fibronectin and integrin-linked kinase 1 (ILK1), and the dysregulation of LATS1-YAP promotes the production of fibronectin and activates ILK1 in PAVSMCs [80, 81]. Selective inhibition of ILK reactivates LATS1 to downregulate YAP, which inhibits proliferation and induces apoptosis of PAVSMCs [80, 81].

**Fig. 3:**
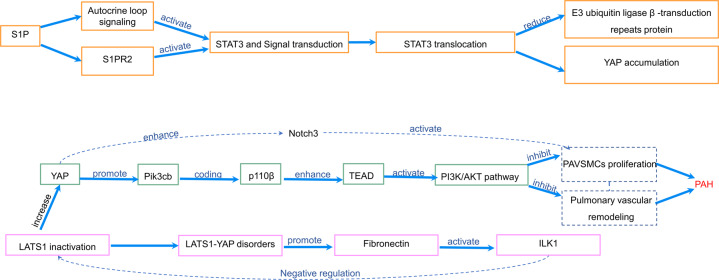
The role of hippo signaling pathway in PAH. Inactivation of LATS1 enhanced the activity of YAP. YAP promotes the transcription of Pik3cb, which encodes the catalytic subunit P110β of PI3K and enhances TEAD, thereby activating the PI3K/AKT pathway. PI3K/AKT pathway promotes PAVSMCs proliferation and pulmonary vascular remodeling and aggravates PAH. In addition, inactivation of LATS1 leads to LATS1-YAP dysregulation, which promotes the production of fibronectin and activates ILK1. Inhibition of ILK reactivating LATS1 in PAVSMCs leads to downregulation of YAP, inhibition of PAVSMCs proliferation, and induction of apoptosis. In addition, S1P promotes the activation of STAT3 through S1PR2 or autocrine loop signaling, which further lead to STAT3 translocates to the nucleus [97, 108]. STAT3 translocation further reduces the expression of E3 ubiquitin ligase β -transduction repeat protein and inhibits degradation of YAP ubiquitination in PAVSMCs. Note: PAH pulmonary arterial hypertension, LATS1/2 large tumor suppressor 1/2, TEAD TEA domain family, S1P sphingosine-1-phosphate, PAVSMCs pulmonary arterial vascular smooth muscle cells, S1PR2 sphingosine-1-phosphate receptor 2, ILK1 integrin-linked kinase 1, PI3K phosphatidylinositol 3-kinase.

In addition, S1P promotes the activation of signal transduction and transcriptional activator 3 (STAT3) through S1PR2 or autocrine loop signaling, which further leads to STAT3 translocating to the nucleus and leads to reduced ubiquitination degradation of YAP in PAVSMCs [82, 83]. Meanwhile, the accumulation of YAP further increases the expression of Notch3, which participates in PAH by promoting PAVSMCs proliferation and pulmonary vascular remodeling [82, 84, 85] (Fig. 3).

### Hippo signaling pathway and pneumonia

Pneumonia refers to inflammation of the terminal airway, alveoli, and interstitium of the lung and is caused by pathogenic microorganisms, immune damage, physical and chemical factors, and allergies [86–90]. Bacterial pneumonia is the most common pneumonia [91].

**Fig. 4:**
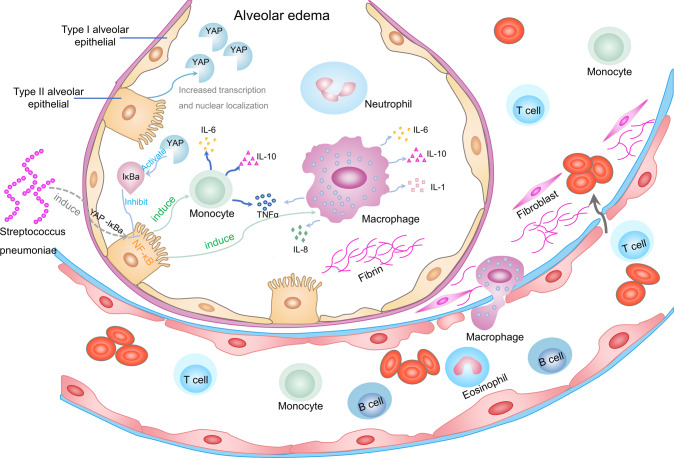
The role of the hippo signaling pathway in pneumonia. S. pneumoniae capsule invades lung tissue and activates NF-κB in AECIIs. NF-κB is involved in regulating lung inflammation by initiating inflammatory mediators, pro-inflammatory cytokines, and chemokines. At this point, YAP activates IκBa which inhibits NF-κB, thereby alleviating the lung inflammation. The loss of YAP in AECIIs promotes the expression of inflammatory genes and the continued accumulation of inflammatory cells, resulting in persistent of lung inflammation and alveolar fibrosis. Note: IL interleukin, AECIIs type II alveolar epithelial cells, AECIs type I alveolar epithelial cells, NF-κB nuclear factor-κB.

The expression and nuclear localization of YAP in AECIIs were significantly increased in mice with bacterial pneumonia, which causes the production of inflammatory cytokines and affects the activation of YAP-IκB [63]. IκB is the inhibitor of NF-κB which, initiates the production of inflammatory mediators, pro-inflammatory cytokines, and chemokines in the alveolar epithelium [92, 93]. YAP alleviates lung inflammation and promotes regeneration of alveolar epithelial cells in bacterial pneumonia by activating IκB [63, 94]. Moreover, the loss of YAP in AECIIs leads to continuous accumulation of inflammatory cells in the lung of bacterial pneumonia, which results in persistent lung inflammation [63] (Fig. 4).

### Hippo signaling pathway and IPF

IPF is a chronic, progressive, age-related, irreversible, fibrotic interstitial lung disease characterized by excessive deposition of extracellular matrix proteins and destruction of alveolar structures [95–99]. Most patients with IPF die of respiratory failure within 3–5 years [97, 99].

Lung fibroblasts are the main effector cells of IPF, and the YAP mediates the proliferation, migration, and collagen deposition of lung fibroblasts induced by mechanical signals [100, 101]. When extracellular matrix stiffness greatens, YAP/TAZ enters into the nucleus and binds to TEAD, which promote transcription of fibrosis Twist1 gene, leading to fibroblasts proliferation, collagen deposition, and change of the fibroblasts from the relatively static state into a state of pathologic activation; thus the pulmonary fibrosis is promoted [38, 39, 74, 102].

Moreover, IPF development is a SMAD-3-dependent process, which increases collagen deposition in AECs. It is reported that YAP promotes the expression of SMAD-3 in pulmonary fibrosis mice [103]. In addition, YAP knockout results in the reduction of cell AECs senescence [103]. Accordingly, the YAP promotes AEC senescence and aggravates the development of IPF.

**Fig. 5:**
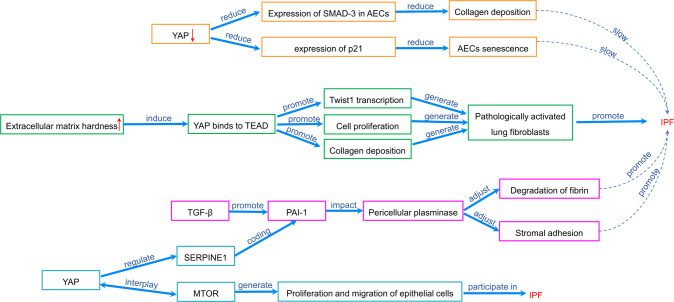
The role of hippo signaling pathway in IPF. When the stiffness of the extracellular matrix increases, YAP enters the nucleus and binds with TEAD to promote Twist1 gene transcription, cell proliferation, and collagen deposition, thus causing pulmonary fibrosis. Meanwhile, TGF-β promotes the expression of PAI-1, which regulates the degradation of fibrin and stromal adhesion of lung fibroblasts by affecting the activity of pericellular plasminase. The Serpine1 gene which encodes PAI-1 is regulated by YAP. In addition, YAP knockout reduces the expression of Smad-3 and p21 in AECs, and reduces collagen deposition and aging AECs, thus slowing down the IPF. YAP can also interact with the mTOR signaling pathway to regulate the abnormal proliferation, polarity and migration of respiratory epithelial cells, and participate in the pathogenesis of IPF. Note: IPF idiopathic pulmonary fibrosis, TEAD TEA domain family, TNF tumor necrosis factor, AECs alveolar epithelial cells, PAI-1 plasminogen activator inhibitor-1, TGF-β transforming growth factor-β, Serpine1 serine protease inhibitor clade E member 1.

Transforming growth factor-β (TGF-β) promotes the expression of plasminogen activator inhibitor-1 (PAI-1), and then PAI-1 promotes fibrosis and regulates degradation of fibrin and stromal adhesion of lung fibroblasts by affecting pericellular plasminase activity, which results in IPF [104]. Serpine1, which encodes PAI-1 is a target gene of YAP/TAZ and is directly regulated by YAP/TAZ [104, 105]. When YAP/TAZ is knocked out, the function of TGF-β is inhibited, which decrease the expression of PAI-1 [104, 105]. Meanwhile, YAP regulates the abnormal proliferation, polarity, and migration of respiratory epithelial cells, inhibits epithelial cell differentiation, and participates in the pathogenesis of IPF through the mTOR signaling pathway [106] (Fig. 5).

### Hippo signaling pathway and asthma

Asthma affects >300 million people around the world and its prevalence is increasing [107–110]. The main features of asthma include airway inflammation, high response of airway smooth muscle to multiple stimuli, and airway remodeling [107, 111–113].

The immune tolerance disorder of Notch4-mediated is a main mechanism that induces chronic inflammation in asthma. It is suggested that the Notch4 causes Treg cell dysfunction through the hippo signaling pathway, which promotes allergic airway inflammation [114]. When alveolar macrophages phagocytose allergens and particulate pollutants, aryl hydrocarbon receptors are activated to induce the expression of Notch ligand Jagged1 (Jag1) which activates Notch on CD4 + T cells, and then Notch transforms Treg cells into Th2 or Th17 effector T (Teff) cells through hippo pathway-dependent mechanisms, thus the stability and function of Treg cells is damaged [114, 115].

**Fig. 6:**
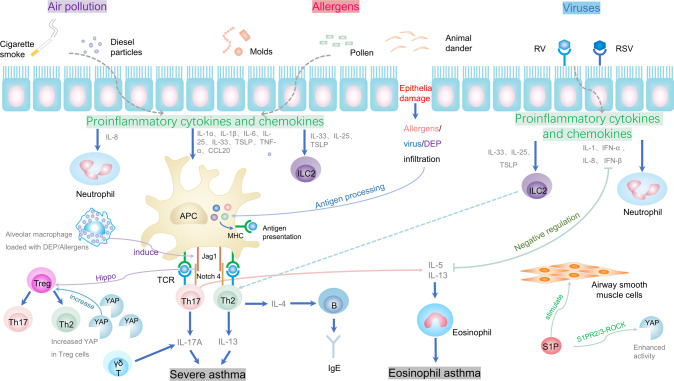
The role of hippo signaling pathway in asthma. Exposure to air pollutants (cigarette smoke, diesel particles, etc.) and allergens (mold, pollen, animal dander, etc.) stimulates epithelial cells leading to the release of pro-inflammatory cytokines (IL-1, IL-6, TNF, etc.) and chemokines (IL-8, CCL20, etc.). Pro-inflammatory cytokines and chemokines act on congenital lymphocytes (ILC2s), neutrophils, and APC. Jag1 on APC binds to Notch receptors on T cells to activate Notch. Notch converts Treg cells into Th2 cells and Th17 cells through hippo pathway-dependent mechanisms, thus damaging the stability and function of Treg cells. Th17 cells and Th2 cells release active mediators, leading to airway smooth muscle contraction, increased viscosity secretion, and inflammatory cell infiltration, which trigger severe asthma. Meanwhile, Th2 cells produce interleukin (IL-4, etc.) to activate B cells to synthesize specific IgE, which binds to IgE receptors on mast cells and eosinophils. If the allergen re-enters the body, it can cross-link with IgE binding to the cell surface, causing the cell to synthesize and release multiple active mediators, producing clinical symptoms of asthma. Interactions of respiratory viruses (RV and RSV) with specific receptors in epithelial cells lead to the release of pro-inflammatory cytokines and chemokines and act on ILC2s and neutrophils, which promote differentiation of Treg cells into IL-13-producing Th2 cells in the absence of allergens. Notch and its downstream effector YAP were significantly increased in Treg cells of patients with severe asthma. Note: RV rotavirus, RSV respiratory syncytial virus, DEP diesel particles, CCL20 CC chemokine ligand 20, TNF tumor necrosis factor, IL interleukin, TSLP thymic stromal lymphopoietin, ILC2s group 2 innate lymphoid cells, APC antigen-presenting cell, Jag1 jagged1, S1P sphingosine-1-phosphate.

Abnormal proliferation and migration of ASMCs play an important role in airway hyperresponsiveness and airway remodeling in asthma [108, 116, 117]. S1P is a natural, multifunctional, bioactive phospholipid molecule that is involved in cell proliferation, differentiation, migration, contraction, and vasculogenesis [82, 108, 118–120]. It is reported that the level of S1P in bronchoalveolar lavage fluid is significantly increased in asthmatic patients, and the S1P stimulates the proliferation, migration, and contraction of ASMC in vitro [108]. Meanwhile, S1P inhibits the phosphorylation of YAP and promotes its nuclear localization [108, 121]. In addition, abnormal YAP in pregnant mice increased the susceptibility of their offspring to asthma [116]. However, the mechanisms by which YAP affects asthma remain unclear (Fig. 6).

## Conclusion

The hippo signaling pathway is mainly composed of MST1/2, LATS1/2, SAV1, MOB1, YAP, or TAZ, and TEAD and is involved in ALI, PAH, pneumonia, IPF, and asthma. YAP/TAZ, as the cascade effector of the hippo signaling pathway, plays an important role in these respiratory diseases. When YAP is activated, YAP transfers from the cytoplasm to the nucleus and accumulates in the nucleus, thereby acting on target genes such as Twist1 and Serpine1. Subsequently, the activation of target genes affects the proliferation and migration of cells such as AECs, PAVSMCs, lung fibroblasts, and AECIIs. However, the specific mechanisms of the hippo signaling pathway regulating respiratory diseases remain unclear and more researches are needed in further.
